# Genetic Analysis of the Measles Virus From the Outbreaks in South Korea, 2019

**DOI:** 10.3389/fmicb.2021.763107

**Published:** 2021-12-15

**Authors:** Jeong-Min Kim, Sehee Park, Sujin Kim, Kye Ryeong Park, Jin-Sook Wang, Yoon-Seok Chung

**Affiliations:** ^1^Division of Emerging Infectious Diseases, Bureau of Infectious Diseases Diagnosis Control, Korea Disease Control and Prevention Agency, Cheongju-si, South Korea; ^2^Division of Acute Viral Diseases, Center for Emerging Virus Research, Korea Disease Control and Prevention Agency, Cheongju-si, South Korea; ^3^Division of Viral Diseases, Bureau of Infectious Diseases Diagnosis Control, Korea Disease Control and Prevention Agency, Cheongju-si, South Korea; ^4^Division of Infectious Disease Diagnosis Control, Honam Regional Center for Disease Control and Prevention, Korea Disease Control and Prevention Agency, Gwangju-si, South Korea

**Keywords:** measles viruses, epidemiology, N-450, reverse transcription-polymerase chain reaction, MF-NCR-H

## Abstract

Three genotypes (B3, D8, and H1) of the measles virus (MeV) have recently caused global outbreaks. In Korea, four measles outbreaks were reported during 2018–2019 and most patients were infants and health care workers in their 20s and 30s. To investigate the genetic characteristics and molecular epidemiology of the outbreaks, we analyzed the sequence of MeVs by targeting the N-450, MF-NCR, and/or H gene regions. Considering their phylogenetic relationships, besides the N-450 and MF-NCR sequences that are commonly used for genotyping MeVs, the MF-NCR-H sequence was related to the dynamics for identifying the transmission of MeVs. Phylogenetic clustering patterns reconstructed from the MF-NCR-H sequence set revealed that genotype D8 caused three of the four outbreaks, while B3 seemed to have induced the fourth outbreak. These results suggest that the MF-NCR-H sequence is useful for rapid confirmation of measles outbreaks and to identify the epidemiological routes of MeVs.

## Introduction

Measles morbillivirus, of the family *Paramyxoviridae*, causes the highly contagious disease, measles, among vulnerable individuals with a broad range of case fatality rates depending on their immunization status, age of infection, and nutrition (Wolfson et al., 2009; Moss, 2017; Minh et al., 2020). Measles can be prevented by two doses of vaccines, and at least 95% vaccination coverage rates are expected to prevent its outbreak (Majumder et al., 2015). However, the first and second dose coverage seems to be approximately 85 and 67%, respectively, (World Health Organization [WHO], 2019b). Based on the provisional surveillance data of the World Health Organization, the reported cases of measles in the first quarter of 2019 increased to approximately 300%, compared to those during the same period in 2018 (World Health Organization [WHO], 2019b). In Korea, the WHO declared the elimination of measles in 2006, probably owing to increased immunization rates through an extensive vaccination program initiated in 2001 (Centers for Disease Control Prevention [CDC], 2007; Muller et al., 2007). However, an increasing number of imported cases of measles by overseas travelers still threatens public health in Korea (Yang et al., 2015; Eom et al., 2018). However, confirmed cases of measles suddenly increased in 2014, and the virus was prevalent again in 2019, resulting in >140 cases in the first 4 months of its prevalence (World Health Organization [WHO], 2019a).

Molecular epidemiology is one of the most important methods to closely investigate evolutionary relationships of individual viral strains and their transmission routes in measles. Furthermore, this approach can help examine nationwide vaccine coverage against measles outbreaks (Rota et al., 1996; Bellini and Rota, 1998). Molecular epidemiology of the measles virus (MeV) can be assessed by detecting the highly variable regions of its single-stranded RNA genome. Considering the genetic variations in the N-450 region, 24 distinct genotypes of MeVs have been identified thus far (No authors listed, 2003; Rota and Bellini, 2003); moreover, the non-coding region (NCR) between matrix (M) and fusion (F) protein genes have been used for genotyping various viral strains, considering their reconstructed phylogenetic relationships (Gardy et al., 2015; Rota and Bankamp, 2015). Of the four main genotypes (B3, D4, D8, and H1), D8 is prevalent in North and South America, Europe, Oceania, and Asia, whereas B3 is widespread in Africa and the Middle East for the past year (as of June 2019) (World Health Organization [WHO], 2019a). Although the genotypes B3 and H1 have caused more outbreaks in China, and genotype B3 has also been reported in Japan, and only D8 has been reported in Korea thus far (World Health Organization [WHO], 2019a).

This study aimed to evaluate the genetic characteristics and molecular epidemiology of the measles outbreaks in Korea, in 2019, by targeting the N-450, MF-NCR, and/or H gene regions. Considering the phylogenetic relationships among N-450, MF-NCR, and MF-NCR-H regions, we subsequently investigated the transmission routes of MeV and discussed the effectiveness and future directions of the national vaccination program against measles in Korea.

## Materials and Methods

### Specimens

A suspected measles case was defined as a patient with fever, maculopapular skin rash, and more than one of the three following symptoms: cough, coryza, or conjunctivitis. Serum, urine, and throat swab samples were obtained from suspected measles for laboratory analysis. For about 6 months from December 2018 to May 2019, the genetic characteristics of the MeVs were analyzed from samples of measles patients identified through the measles monitoring system in South Korea.

### Immunoassay

Measles-specific IgM and IgG antibodies were detected using commercial ELISA kits (Enzygnost anti-measles Virus/IgM and/IgG, respectively, Siemens Healthcare Diagnostics, Germany) in accordance with the manufacturer’s instructions. The results were classified as follows: optical density > 0.2 was positive, 0.1–0.2 was equivocal, and < 0.1 was negative. IgG titer was calculated, and ODs were converted to international units using the α-method in accordance with the manufacturer’s instructions. A fourfold increase in the IgG titer of convalescent serum (collected 10–30 days after the collection of acute serum) compared to that of acute serum was determined compatible with recent measles infection.

### Real-Time RT-PCR for MeV Detection

Real-time RT-PCR assays were performed for the detection of measles virus N gene using the 7,500 fast Real-time PCR system (Applied Biosystems). The amplification used a forward (MVN1139-F: 5′-TGGCATCTGAACTCGGTATCA C-3′) and a reverse (MVN1213-R: 5′-TGTCCTCAGT AGTATGCATTGCAA-3′) primer. A probe (MVNP1163-P: 5′-CCGAGGATGCAAGGCTTGTTTCAGA-3′) was labeled at the 5′ terminus with a fluorescent reporter dye, 6-carboxyfluorescein (FAM), and at the 3′ terminus with a non-fluorescent quencher, black hole quencher-1 (BHQ1). The amplification conditions were as follows: 50°C for 30 min, followed by 95°C for 10 min and 40 cycles of 95°C for 15 s and 60°C for 1 min. Real-time RT-PCR assays has been verified and used by the Standard Operation Protocol (SOP) Verification Committee in Korea Disease Control and Prevention Agency (KDCA).

### Genotype Identification and Genetic Analysis

Viral RNAs were extracted from throat swab samples using a QIAamp Viral RNA Mini Kit (Qiagen, Venlo, Netherlands) in accordance with the manufacturer’s instructions. The highly variable 450-nucleotide (nt) region in the carboxy-terminus of nucleocapsid protein (N-450) was amplified and sequenced for genotyping using forward (MeV216: 5′-TGGAGCTATGCCATGGGAGT-3′) and reverse (MeV214: 5′-TAACAATGATGGAGGGTAGG-3′) primers. RT-PCR was performed using OneStep RT-PCR Kit (Qiagen, Venlo, Netherlands) in accordance with the manufacturer’s instructions. The amplification conditions were as follows: 50°C for 30 min, followed by 95°C for 15 min, and 40 cycles of 95°C for 30 s, 95°C for 30 s, and 72°C for 30 s, with a final 10-min extension at 72°C.

cDNAs between matrix (M) gene end and Hemagglutinin (H) gene end (MF-NCR-H) were obtained with the primer MeH6_R: 5′-CAGATAGCGAGTCCATA ACG-3′ using SuperScript III reverse transcriptase (Invitrogen, Carlsbad, CA, United States) in accordance with the manufacturer’s instructions. PCR was performed using appropriate forward (MeV4200_F: 5′-GGCACCAGTCTTCACATYAGAAG-3′) and reverse (MS9221_R: 5′-CTTGGACCCTAYCTTTTTCTTAAT-3′) primers. The amplification conditions were as follows: 94°C for 3 min, followed by 35 cycles of 94°C for 30 s, 58°C for 30 s, and 72°C for 5 min, finally ending with an extension at 72°C for 10 min. we sequenced the resulting PCR amplicons by Sanger sequencing using an ABI 3730 Analyzer (Applied Biosystems)^1^.

### Phylogenetic Analysis

The sequences obtained herein were aligned with the CLC Main Workbench 7.9.1, including genotypes D8 and B3 reference sequences from GenBank. MEGA6 was used to generate phylogenetic trees through the neighbor-joining method using the maximum composite likelihood-parameter distance matrix listed in the software; bootstrap values were obtained through random sampling of 1,000 replicates.

## Results

### Population Analysis of the Patients Diagnosed With Measles and Laboratory Diagnosis

**FIGURE 1:**
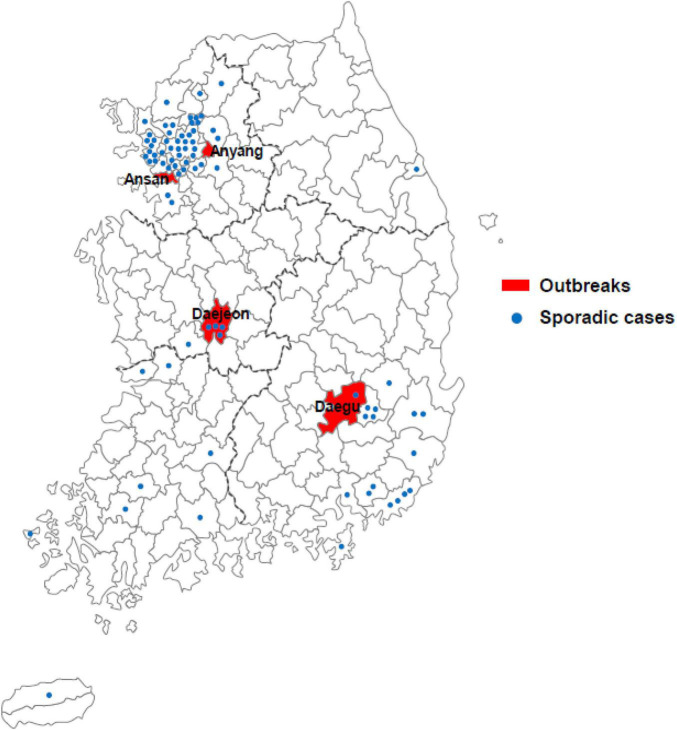
Geographical distribution of cases of measles across South Korea in 2019. Measles outbreaks occurred in four regions of South Korea (Daegu, Ansan, Anyang, and Daejeon; red fill). Sporadic cases are represented by blue dots.

In South Korea, 4 large outbreaks (Daegu, Ansan, Anyang, and Daejeon) had occurred, along with sporadic cases, from December 2018 to May 2019, with 163 confirmed cases (Figure 1). Measles cases identified as measles with specific IgM antibodies (anti-MeV IgM) and measles virus N gene detection (MeV RNA) of measles viruses were analyzed using ELISA and real-time RT-PCR. Among the 163 patients, 161 (98.8%) were detected with MeV RNA, and 49 (30.0%) had anti-MeV IgM. In term of sex, 94 (57.7%) were female and 69 (42.3%) were male. The age group accounting for the largest proportion was of 20–29 years (41.7%). Most confirmed cases in the outbreak group in Anyang were health care workers, most being female nurses aged 20–29 years of age. Forty-five of 163 patients (27.6%) were up to 4 years old; furthermore, in a pediatric hospital in Daejeon, 10 of 20 patients (50.0%) were infants under 12 months of age. In South Korea, genotypes B3 and D8 were identified between December 2018 and May 2019, and no case of the endemic genotype (H1) was noted. The number of imported cases with a documented travel history was 61 (37.4%). The index case of three outbreaks (Daegu, Ansan, and Anyang) is unknown, although genetic analysis confirmed it to have been imported from abroad (Table 1).

**TABLE 1:** Characteristics of 163 confirmed cases of measles in South Korea, December 2018–May 2019.

Characteristics	Daegu (*n* = 16%)	Ansan (*n* = 22%)	Anyang (*n* = 26%)	Daejeon (*n* = 20%)	Sporadic (*n* = 79%)	Total (*n* = 163%)
**Diagnosis^a^**						
Real-time RT-PCR	9 (56.2)	15 (68.2)	22 (84.6)	12 (60.0)	56 (70.9)	114 (70.0)
IgM	1 (6.3)	0 (0.0)	0 (0.0)	0 (0.0)	1 (1.3)	2 (1.2)
Real-time RT-PCR/IgM	6 (37.5)	7 (31.8)	4 (15.4)	8 (40.0)	22 (27.8)	47 (28.8)
**Age group, y**						
< 1	6 (37.5)	4 (18.2)	0 (0.0)	10 (50.0)	5 (6.3)	25 (15.3)
1–4	2 (12.5)	7 (31.9)	0 (0.0)	5 (25.0)	6 (7.6)	20 (12.3)
5–9	0 (0.0)	0 (0.0)	0 (0.0)	0 (0.0)	0 (0.0)	0 (0.0)
10–14	0 (0.0)	0 (0.0)	0 (0.0)	0 (0.0)	3 (3.8)	3 (1.8)
15–19	0 (0.0)	0 (0.0)	1 (3.8)	0 (0.0)	6 (7.6)	7 (4.3)
20–24	5 (31.3)	3 (13.6)	17 (65.4)	1 (5.0)	19 (24.0)	45 (27.6)
25–29	0 (0.0)	4 (18.2)	8 (30.8)	2 (10.0)	9 (11.4)	23 (14.1)
30–34	1 (6.3)	1 (4.5)	0 (0.0)	1 (5.0)	7 (8.9)	10 (6.1)
≥ 35	2 (12.5)	3 (13.6)	0 (0.0)	1 (5.0)	24 (30.4)	30 (18.4)
**Sex**						
Female	11 (68.8)	14 (63.6)	20 (76.9)	11 (55.0)	38 (48.1)	94 (57.7)
Male	5 (31.2)	8 (36.4)	6 (23.1)	9 (45.0)	41 (51.9)	69 (42.3)
**Vaccination status^b^**						
1 dose	3 (18.8)	1 (4.5)	17 (65.4)	7 (35.0)	12 (15.2)	40 (24.5)
2 doses	5 (31.2)	4 (18.2)	7 (26.9)	3 (15.0)	8 (10.1)	27 (16.6)
Unvaccinated	5 (31.2)	9 (40.9)	0 (0.0)	9 (45.0)	9 (11.4)	32 (19.6)
Unknown	3 (18.8)	8 (36.4)	2 (7.7)	1 (5.0)	50 (63.3)	64 (39.3)
**Infection source**						
Imported	0 (0.0)	0 (0.0)	0 (0.0)	1 (5.0)	60 (75.9)	61 (37.4)
Import-related	16 (100.0)	22 (100.0)	26 (100.0)	19 (95.0)	16 (20.3)	99 (60.7)
Unknown	0 (0.0)	0 (0.0)	0 (0.0)	0 (0.0)	3 (3.8)	3 (1.8)
**Genotype**						
B3	14 (87.5)	0 (0.0)	0 (0.0)	0 (0.0)	26 (32.9)	40 (24.5)
D8	0 (0.0)	22 (100.0)	26 (100.0)	20 (100.0)	47 (59.5)	115 (70.6)
Unknown*^c^*	2 (12.5)	0 (0.0)	0 (0.0)	0 (0.0)	6 (7.6)	8 (4.9)

*^a^Real-time RT-PCR refers to the detected measles virus gene, and not detected or have not specimen for measles virus-specific IgM antibodies. IgM refers to detected measles virus-specific IgM antibodies and have not specimen for measles virus gene. Real-time RT-PCR/IgM refers to detected measles virus gene as well as measles virus-specific IgM antibodies.*

*^b^First dose of measles containing vaccine at 12–15 months of age and second dose at 4–6 year of age.*

*^c^Among the 8 patients, 2 were confirmed as IgM-positive and have not specimen for measles virus gene, 6 were though measles virus detected using real-time RT-PCR, genotyping was not confirmed.*

### Genetic and Phylogenetic Analyses

**FIGURE 2:**
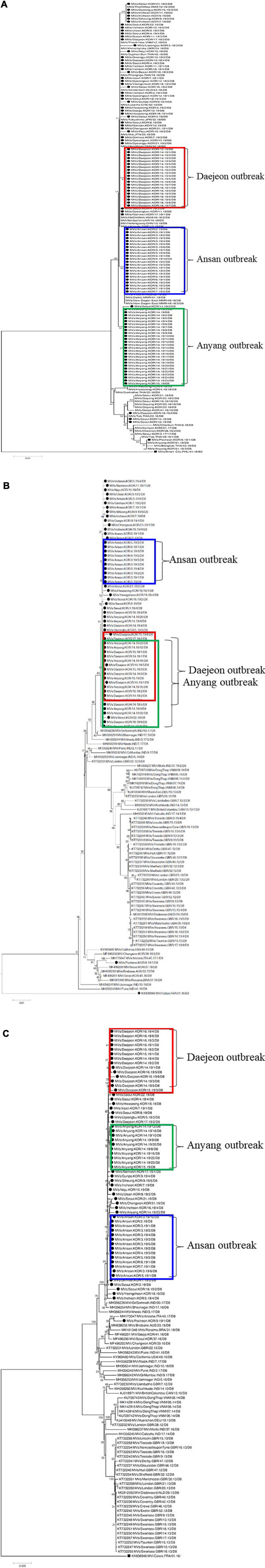
Phylogenetic analysis of MeV genotype D8 strains based on nucleotide sequences using the neighbor-joining tree. Values on branches are shown as percentages on the basis of 1,000 bootstrap replicates. The black dot indicates the MeV genotype D8 relevant to Korean occurrences in 2019 and named strains accepted in MeaNS. The tree was rooted with respect to the genotypes D8 and B3 based on WHO and GenBank reference sequence. **(A)** N-450 gene analysis of MeV genotype D8. The black squares indicate the MeV genotype B3 reference using genotype D8 outgroup. **(B)** MF NCR gene analysis of MeV genotype D8. The black squares indicate the MeV genotype D8 reference using genotype B3 outgroup. **(C)** MF NCR-H gene analysis of MeV genotype D8. The black squares indicate the MeV genotype D8 reference using genotype B3 outgroup. The blue box indicates Ansan, the green box indicates Anyang, and the red box indicates Daejeon, respectively.

Analysis of the 155 sequences of MeV N-450 gene and 81 sequences of MeV MF-NCR-H gene identified 115 and 60 sequences of the D8 genotype and 40 and 21 sequences of the B3 genotype, respectively. Three of these cases are known strains in the MeaNS database, and belonged to the same genotype, in accordance with the topology of the phylogenetic tree. In case of MeV D8 genotype, the identified N-450 gene sequence cluster was confirmed in a phylogenetic clade of the Daejeon, Ansan, and Anyang outbreaks (Figure 2A). The identified MF-NCR gene sequences clustered in the phylogenetic clade of Ansan, whereas those of Daejeon and Anyang outbreaks are not indicated. Interestingly, phylogenetic analysis of the MF-NCR-H gene sequence revealed consistency with a consequence of the N-450 gene (Figure 2B). According to the MeaNS database, the Daejeon outbreak was exactly matched with Vietnam strain and Ansan outbreak exactly matched with the Myanmar strain; furthermore, the Anyang outbreak was also related to the Vietnam strain. The sporadic cases, excluding the 3 outbreaks, were identified with individual import cases.

**FIGURE 3:**
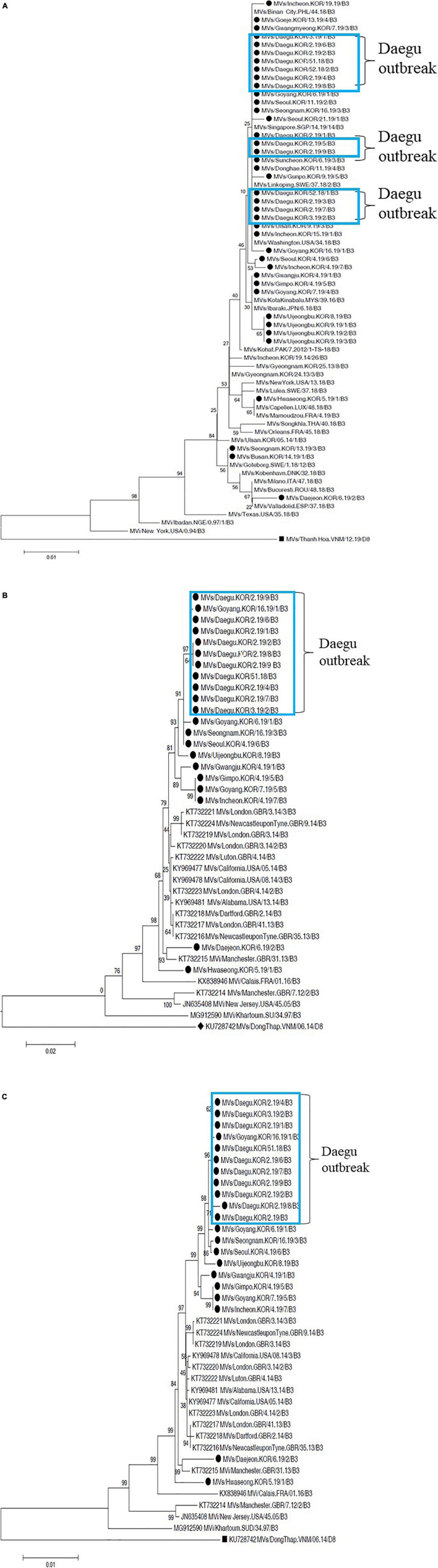
Phylogenetic analysis of MeV genotype D8 strains based on nucleotide sequences using the neighbor-joining tree. Values on branches are shown as percentages on the basis of 1,000 bootstrap replicates. The black dot indicates the MeV genotype D8 relevant to Korean occurrences in 2019 and named strains accepted in MeaNS. The tree was rooted with respect to the genotypes D8 and B3 based on WHO and GenBank reference sequence. **(A)** N-450 gene analysis of MeV genotype D8. The black squares indicate the MeV genotype B3 reference using genotype D8 outgroup. **(B)** MF NCR gene analysis of MeV genotype D8. The black squares indicate the MeV genotype D8 reference using genotype B3 outgroup. **(C)** MF NCR-H gene analysis of MeV genotype D8. The black squares indicate the MeV genotype D8 reference using genotype B3 outgroup. The skyblue box indicates Daegu outbreak.

The Daegu outbreak identified with MeV B3 genotype, and matched with all cases of the N-450 and MF-NCR-H gene sequences clustered in a phylogenetic clade and exactly matched with Philippines strain based on the MeaNS database. The sporadic cases were identified with individual imported cases, together with the MeV D8 genotype (Figure 3).

Finally, the nucleotide sequence homology comparison between N-450 of D8 and B3 identified 100% homology within the outbreak. The MF-NCR and MF-NCR-H genes were identified within a range of 97.54–100 and 99.69–100%, respectively (Table 2).

**TABLE 2:** Comparison of the similarity of the identified measles genes in South Korea from December 2018 to May 2019.

Gene (%)	Daegu	Ansan	Anyang	Daejeon
N-450	100	100	100	100
	(*n* = 14)	(*n* = 22)	(*n* = 26)	(*n* = 20)
MF NCR	99.90–100	99.80–100	99.80–100	97.54–100
	(*n* = 10)	(*n* = 13)	(*n* = 10)	(*n* = 14)
MF NCR-H	99.81–100	99.89–100	99.85–100	99.69–100
	(*n* = 10)	(*n* = 13)	(*n* = 10)	(*n* = 14)

## Discussion

Since the declaration of the elimination of measles by WHO in 2006, recurrent outbreaks have been sporadically reported in Korea (Choe et al., 2013). Especially in 2007, 2010, 2013, and 2014, > 100 cases have been reported, and even in the first three quarters of 2019, 352 cases have been confirmed till date (as of September 8, 2019), which has been the second highest since 2002 (Centers for Disease Control Prevention [CDC], 2007). Introduction of a single virus from overseas might instigate some index cases, after which the virus spreads very efficiently across vulnerable individuals to cause multiple outbreaks in Korea (Eom et al., 2018). The levels of existing neutralizing antibodies among vaccinated individuals appear to largely affect the outbreak size and duration. Hence, gradual waning of measles antibodies after vaccination must be considered for implementing an optimized vaccination program against measles (Kang et al., 2017). Of note, the latest measles outbreaks in Korea was reported among unvaccinated infants in nursery centers and health care workers (in their 20s or 30s with waning the measles antibody) across hospitals (Table 1), in accordance with the 2014 immunodeficiency survey.

As recently reported, the world has also faced a recurrence of measles since 2006. In particular, the number of measles patients surged worldwide over the first half year in 2019, and among the five genotypes currently in circulation (B3, D3, D9, G3, and H1), the D8 genotypes were mainly found in Europe and Asia, while the B3 and H1 genotypes were dominant in the United States and China, respectively (World Health Organization [WHO], 2019a). In Korea, most cases reported in 2015 seemed to have been caused by the genotype D8. However, considering the global economic and human networks, any genotype could be newly introduced in Korea, and the genotype(s) with better viral fitness, in terms of viral transmission and/or immune evasion from vaccine-induced antibodies, potentially replacing the old ones. Considering the unpredictability of measles outbreaks, rapid detection of measles cases and analysis of their genealogical relationships by comparing with previous outbreaks would be essential. Globally, only four genotypes (B3, D4, D8, and H1) appear to be primarily circulating, of which D8 is responsible for > 80% of human cases (World Health Organization [WHO], 2019a). It was the same in Korea, and MeV strains of the three Korean outbreaks (Daejeon, Ansan, and Anyang) in 2018–2019 were associated with genotype D8 (Figure 1); one outbreak (Daegu) was caused by the genotype B3 (Figure 2). As presented in Figures 2, 3, by using MF-NCR-H, not just N-450, we could interpret how different Korean measles outbreaks were related each other in a genomic level. In Figure 2A, which was obtained only using the N-450 sequences, Daejeon outbreak might be closely related to Ansan outbreak. However, the MF-NCR-H sequences of Korean measles outbreaks suggest the close relatedness of Daejeon and Anyang outbreaks. It is similarly demonstrated in Figure 3. In Figure 3A, the Daegu sequences appeared to be dispersed along with the sequences of other regions. In Figure 3B, however, by analyzing the MF-NCR-H sequences, we could observe the closely grouped Daegu sequences together whereas N-450 sequence analysis resulted in the dispersed Daegu sequences with others. These might indicate the usefulness of the MF-NCR-H sequences of measles virus for investigating the molecular epidemiology of measles outbreaks. Furthermore, given the grouping patterns of the reference sequences along with the Korea measles sequences, our phylogenetic analysis suggested Philippines-strain possibly to be the source of one of the Korean outbreaks in 2018–2019 even though traveling routes of the index patients in the Anyang and Daegu outbreaks were not well-matched with their genetic relatedness to Vietnam- and Philippines-like strains.

The genetic identification and characterization of reporting measles viruses enabled us to highlight the specific occurrence of four different outbreaks caused by two genotypes (D8 and B3) in the Republic of Korea. However, this molecular epidemiological study required a lot of comparable genomic reference information. In the case of the Measles virus, there were limitations in sufficient comparison and analysis of infection sources due to very few country-specific genetic information of MF-NCR-H sequences. In addition, MF-NCR-H sequences cannot have sequences in all samples due to differences in the sensitivity of primers. Therefore, continuing international imports of the measles virus and the production and storage of genomic information of outbreaks in the event of a resurgence are especially important.

In conclusion, molecular epidemiology, phylogenetic analysis, and the study of the transmission clusters could be considered important tools to maintain the level of measles elimination. In fact, this multi-faceted approach enables us to track the introduction of imported strains, to observe their persistence in a defined geographic area and highlight the occurrence of large epidemics and their periodic patterns.

## Data Availability Statement

The analysis of the genotypes in this study has been supported by the use of MeaNS database and sequences have been shared with the WHO community. The sequences obtained in this study have been deposited in GenBank with the accession numbers MN845930-MN845950 and MN863736-MN863796 for M-F NCR, F, and H genes.

## Ethics Statement

This study was approved by the Korea Centers for Diseases Control and Prevention Ethics Committee—KCDC Authority (approval number # 2016-10-02-C-A). The requirement for informed consent was waived by Korea Centers for Diseases Control and Prevention Research Ethics Committee as this study was part of a public health surveillance and outbreak investigation in Republic of Korea. This study was performed in accordance with the relevant laws and regulations that govern research in the Korea Centers for Diseases Control and Prevention. Written informed consent for participation was not required for this study in accordance with the national legislation and the institutional requirements.

## Author Contributions

J-MK, SP, and Y-SC conceived this study. J-MK, SP, SK, KP, J-SW, and Y-SC performed measles sequencing and bioinformatics analysis. J-MK and Y-SC conducted phylogenetic tree analysis and global measles sequence analysis. All authors wrote the manuscript.

## Conflict of Interest

The authors declare that the research was conducted in the absence of any commercial or financial relationships that could be construed as a potential conflict of interest.

## Publisher’s Note

All claims expressed in this article are solely those of the authors and do not necessarily represent those of their affiliated organizations, or those of the publisher, the editors and the reviewers. Any product that may be evaluated in this article, or claim that may be made by its manufacturer, is not guaranteed or endorsed by the publisher.

## Footnotes

1
http://www.thermofisher.com
